# Bridging Pyroptosis and Immunity: A Comprehensive Study of the Pyroptosis-Related Long Non-Coding RNA Signature in Breast Cancer

**DOI:** 10.3390/life13071599

**Published:** 2023-07-21

**Authors:** Ye Tian, Jing Dong, Lin Li

**Affiliations:** 1 College of Animal Science and Veterinary Medicine, Shenyang Agricultural University, Shenyang 110866, China; 2020200160@stu.syau.edu.cn; 2 Key Laboratory of Livestock Infectious Diseases, Ministry of Education, Shenyang Agricultural University, Shenyang 110866, China

**Keywords:** pyroptosis, LncRNAs, breast cancer, lncRNA, tumor immune microenvironment

## Abstract

Breast cancer continuously poses serious clinical challenges to human health due to its intrinsic heterogenicity and evolving drug resistance. Recently, increasing evidence has shown that pyroptosis, known as a programmed and inflammatory form of cell death, participates in tumorigenesis, progression, and remodeling of the tumor immune microenvironment (TIME). However, a comprehensive insight into pyroptosis-related signatures for breast cancer remains elusive. The current study established a pyroptosis-related lncRNA signature using transcriptome data and corresponding clinical information from The Cancer Genome Atlas (TCGA). Pyroptosis-related gene clusters, the associated differential expression in breast cancer patients’ subtypes, and the potential mechanisms were all discussed. This integrative analysis revealed a unique signature underpinning the dichotomy of breast cancer progression and survival outcomes. Interestingly, the pyroptosis-related lncRNA signature was revealed as closely intertwined with the TIME. A correlation was established between the pyroptosis-related LncRNA signature and the TIME, underlying the mutual effect between pyroptosis and the immune responses implicated in breast cancer. The findings in this work underline the critical role exerted by pyroptosis in breast cancer, providing new insights into disease progression, prognosis, and therapeutic potential. This work has been poised to provide new avenues for personalized, immune-based cancer therapeutics by enhancing our understanding of pyroptosis in breast cancer.

## 1. Introduction

As the Global Cancer Statistics 2020 indicates, breast cancer (BC) has become the most common type of cancer reported globally and is the leading cause of cancer-related fatalities among women [1]. Breast cancer is highly diverse in its morphology, phenotype, and molecular structure [2]. The primary basis for its histological categorization lies in the expression of various hormone receptors, including human epidermal growth factor receptor 2 (HER2), estrogen receptors (ERs), and progesterone receptors (PRs), in addition to the proliferation marker Ki-67. Depending on the presence or absence of these molecular markers, there are four principal types of breast cancer: HER2+ subtype, non-luminal (characterized by ER+ and/or PR+, HER2+), HER2+ subtype, luminal (characterized by ER−, PR−, HER2+), luminal A-like subtype (ER+ and/or PR+, HER2−, Ki-67 < 14%), luminal B-like subtype (ER+ and/or PR+, HER2+ or HER2−, Ki-67 > 14%), and basal-like subtype (triple-negative breast cancer, TNBC) (ER−, PR−, HER2−) [3,4]. Due to the heterogenicity of BC, a more precise classification of its subtypes and their corresponding individualized treatments are in urgent need [5]. Recent emerging studies have shed light on the pivotal role of programmed cell death, especially for pyroptosis, in cancer biology [6,7,8].

Pyroptosis is a kind of inflammatory programmed cell death induced by activating caspases and gasdermin proteins, ultimately leading to cell perforation, cell lysis, and the release of inflammatory molecules, like IL-18 and IL-1β [9]. Pyroptosis was initially discovered in 1992 through the process of the apoptosis of macrophages induced by *Shigella flexneri* [10] and was subsequently distinguished from apoptosis due to the release of IL-1β along with the destruction of cell membrane integrity [11,12,13]. Pyroptosis is mainly represented as the canonical and the non-canonical pathways. In the canonical pathway, caspase-1 is activated by inflammasomes responding to damage-associated molecular patterns (DAMPs) and pathogen-associated molecular patterns (PAMPs) sensed by cytosolic pattern recognition receptors (PRRs), and then cleaves GSDMD, precursor IL-18, and precursor IL-1β, leading to cell pyroptosis [14,15,16]. In the non-canonical pathway, caspase-4/5/11 is activated by direct lipopolysaccharide (LPS) binding, cleaving GSDMD and initiating pyroptosis. In this pathway, the precursors IL-18 and IL-1β are activated by NLPR3/caspase-1 [17,18]. In addition, pyroptosis can also be induced by the caspase-3/GSDME pathway, granzyme A (Gzm A), granzyme B (Gzm B), and streptococcal pyrogenic exotoxin B (SPEB) [19,20,21,22].

In recent years, researchers have revealed that pyroptosis exerts an essential and dual effect on various aspects of cancer progression, including proliferation, invasion, and metastasis [23,24]. The initiation of pyroptosis in tumor cells results in their lytic death, thereby directly suppressing the growth and proliferation of breast cancer [25]. This suggests that the induction of pyroptosis may serve as a fresh therapeutic approach for breast cancer. Nevertheless, pyroptosis, as a form of pro-inflammatory programmed cell death, causes the breakdown of the cell membrane along with the release of intracellular cytokines, such as IL-1β and IL-18, potentially leading to local and general inflammation [26]. Studies have reported that a heightened level of IL-1β in serum is found in breast cancer patients and is associated with advanced stages and a poorer prognosis [27,28,29]. In addition, advanced-stage breast cancer patients have also been observed to have increased serum levels of IL-18 [30,31]. GSDMB, which is one of the executors of pyroptosis, acts as an oncogene, and the over-expression of GSDMB is responsible for exerting resistance to chemotherapy [32,33]. Interestingly, in an immunohistochemical study on breast cancer, upregulated GSDMD levels were found to have been associated with a lower historical grade and historical stage, indicating that GSDMD acts as a tumor-suppressor gene in breast cancer [34]. However, despite these advances, a comprehensive understanding of the pyroptosis-related signature in BC and its implications for patient prognosis remains elusive.

The tumor microenvironment (TME), which involves tumor cells, various stromal and immune cells, the extracellular matrix, and cytokines, significantly influences cancer development, progression, and therapy responses [35]. Pyroptosis has been reported to dynamically modulate the TME, influencing tumor progression and treatment responses [36,37]. Inflammatory cytokines, such as IL-1β and IL-18, and damage-associated molecular patterns (DAMPs) released during pyroptosis can reconfigure the immune microenvironment, activate tumor immunity, or even confer beneficial effects on tumorigenesis and cancer progression [38,39,40].

As a kind of regulatory non-coding RNA with a length of 200 nt and more, lncRNAs play a crucial part in various steps of the tumorigenesis process, such as angiogenesis, invasion, metastasis, and proliferation [41,42,43]. LncRNAs are dysregulated in BC, leading to increasing studies on the prognostic and therapeutic potential of lncRNAs in BC [44,45,46]. Particularly, lncRNAs have been reported to modulate pyroptosis, thereby contributing to tumorigenesis and progression [47,48]. Intriguingly, it has been suggested by accumulating evidence that pyroptosis can reshape the tumor immune microenvironment, implying that lncRNAs possibly influence the immune landscape of breast cancer by regulating pyroptosis. However, the roles of lncRNAs associated with pyroptosis in breast cancer and their underlying regulatory mechanisms remain largely unexplored.

Competing endogenous RNAs (ceRNAs), initially proposed by Salmena and colleagues in 2011, was described as a complex regulatory network where mRNAs, lncRNAs, and other RNA species competed for the shared microRNA (miRNA) response elements (MREs) [49]. In particular, lncRNAs have been shown to act as ceRNAs, sequestering miRNAs, and subsequently influencing the expression of target mRNAs in the regulation of pyroptosis, which exerts a crucial effect on cancer progression and the TME [50,51,52].

Considering the significance of pyroptosis and the TME in breast cancer, our study used TCGA data to dissect the pyroptosis-related lncRNA signature and explored the associated ceRNA network. This comprehensive investigation offered innovative views into the lncRNA-mediated regulation of pyroptosis in breast cancer, potentially providing a new avenue for therapeutic intervention and prognostic assessment.

In light of these developments, the current work sought to identify a pyroptosis-related lncRNA signature in breast cancer using TCGA data, investigate its associated TME, and elucidate their associated ceRNA network. The results of our study aimed to shed light on how the lncRNA-induced regulation of pyroptosis impacts breast cancer, potentially paving the way for personalized treatments and aiding in the prognosis assessment.

## 2. Materials and Methods

### 2.1. Data Collection

In total, the transcriptional data of 1060 tumor samples and 111 normal samples, as well as clinical data and single nucleotide variation data were downloaded from TCGA database (https://portal.gdc.cancer.gov).

### 2.2. Screening Pyroptosis-Related Differentially Expressed Genes (DEGs) and Analyzing Their Functions

DEGs were screened using the “limma” package (Version 3.55.7) with the threshold of |log_2_FC| ≥ 0.585 and FDR < 0.05. The R packages “ClusterProfiler” (Version 4.8.1), “enrichplot” (Version 1.20.0), and “ggplot2” (Version 3.4.2) were used to conduct Kyoto encyclopedia of genes and genomes (KEGG) analysis and gene ontology (GO) functional analysis. The interaction between these pyroptosis-related DEGs was analyzed using STRING Version 11.5 (https://string-db.org/) with a threshold interaction score of 0.4 [53,54,55,56,57,58].

### 2.3. The Identification of Pyroptosis-Related LncRNAs

Pyroptosis-related lncRNAs were identified through conducting Pearson’s correlation analysis of the pyroptosis-related genes with the correlation coefficient threshold >0.3 and *p* < 0.05.

### 2.4. The Establishment and Verification of the Pyroptosis-Related LncRNAs Signature

Pyroptosis-related differentially expressed lncRNAs (DElncRNAs) were screened using the “limma” package with the threshold |log_2_FC| ≥ 1 and FDR < 0.05. The result was visualized using the “pheatmap” package (Version 1.0.12).

The prognostic model was established and visualized using R packages “survival” (Version 3.5-5), “caret” (Version 6.0-94), “glmnet” (Version 4.1-7), “survminer” (Version 0.4.9), “pheatmap”, and “timeROC” (Version 0.4). The whole dataset was randomly divided into a train group and a test group, and a univariate Cox analysis of overall survival (OS) was performed on the pyroptosis-related DElncRNAs with a *p*-value of 0.05. To establish the prognostic model, lasso regression and multivariate Cox analysis were successively conducted on the significant candidate DElncRNAs obtained from the univariate Cox analysis [59,60,61,62,63,64].

Patients were divided into a high-risk group and a low-risk group according to the median value of their risk scores. Kaplan–Meier (K–M) analysis was performed on the two groups’ overall survival (OS). Univariate and multivariate independent prognostic analyses were conducted and then visualized in forest plots. The prognostic potential of the pyroptosis-related lncRNA signature was evaluated using the “time ROC” package. A nomogram and its corresponding calibration curve were drawn to predict the OS using age, stage, risk score, and TMN classification.

### 2.5. Screening Risk-Related DEGs and Analyzing Their Functions

The DEGs between the categorized high-risk and low-risk groups were screened using the “limma” package with the threshold |log_2_FC| ≥ 1 and FDR < 0.05. GO and KEGG analyses were performed using the R packages “enrichplot” and “ggplot2” [57].

### 2.6. Gene Set Enrichment Analysis (GSEA) of the High- and Low-Risk Groups

GSEA was conducted and visualized using the “ClusterProfiler” and “enrichplot” packages using the gene set c2.cp.kegg.Hs.symbols.gmt with a threshold of *p*-value < 0.05 [54,55,65,66].

### 2.7. Tumor Mutation Burden (TMB) of the High- and Low-Risk Groups

TMB of the high-risk and low-risk groups was analyzed and then visualized using the R package “maftools” (Version 2.16.0). Survival analyses of high and low TMB and the combination of the TMB and risk were performed and visualized using the “survival” and “survminer” packages [67].

### 2.8. The Tumor Immune Microenvironment of the High- and Low-Risk Groups

The TME scores of the high- and low-risk groups were calculated and visualized with the R packages “limma” and “ggpubr” (Version 0.6.0). The correlation between immune cell infiltration and risk was analyzed and visualized with the R packages “limma”, “ggtext” (Version 0.1.2), “ggplot2”, “scales” (Version 1.2.1), “reshape2” (Version 1.4.4), “tidyverse” (Version 2.0.0), and “ggpubr” using seven software tools, including EPIC, CIBERSORT, CIBERSORT-ABS, QUANTISEQ, XCELL, MCPCOUNTER, and TIMER. Patients were divided into high and low groups on the basis of their immune cell contents calculated by EPIC, CIBERSORT, CIBERSORT-ABS, QUANTISEQ, XCELL, MCPCOUNTER, and TIMER. Survival analyses were performed and visualized with the “survival”, “limma”, and “survminer” packages. Single-sample GSEA (ssGSEA) was conducted using the “GSVA” (Version 1.48.1), “limma”, and “GSEABase” packages (Version 1.62.0). The immune cell contents and functions of the high- and low-risk groups were analyzed and visualized using the “limma”, “ggpubr”, and “reshape2” packages. The expressions of the immune checkpoint proteins of the high- and low-risk groups were analyzed and visualized with the “limma”, “ggpubr”, “ggplot2”, and “reshape2” packages. The drug sensitivity of the high- and low-risk groups was analyzed using the R packages “limma”, “oncoPredict” (Version 0.2), and “parallel” (Version 3.6.2), and then visualized with the “ggpubr” and “ggplot2” packages [68,69,70,71,72,73,74,75,76,77,78].

### 2.9. Cluster of Breast Cancer Subtypes and Their Associated Functional Analysis

The patients in this work were clustered into three subtypes on the basis of their pyroptosis-related lncRNA signature and risk score using the R packages “limma” and “ConsensusClusterPlus” (Version 1.64.0). As previously mentioned, the survival analysis of these three subtypes was performed and visualized. The relationship between these three subtypes and the high- and low-risk groups was analyzed and visualized with the “ggalluvial” (Version 0.12.5), “ggplot2”, and “dplyr” packages (Version 1.1.2). PCA and t-SNE analyses of these three subtypes and of the high- and low-risk groups were performed and visualized using the R packages “ggplot2” and “Rtsne” (Version 0.16). The TME scoring, contents of various immune cell species, checkpoint gene expressions, and drug sensitivity between subtypes were analyzed and visualized, as previously mentioned [79,80,81,82].

### 2.10. The Construction of the ceRNA Regulatory Network

The target miRNAs of the pyroptosis-related DElncRNAs and the pyroptosis-related DEGs were predicted in the miRcode database, respectively (miRcode—transcriptome-wide microRNA target prediction including lncRNAs). Potential target genes of the intersection miRNAs were screened from 3 databases, including TargetScan (TargetScanHuman 7.1), miRTarBase (miRTarBase: the experimentally validated microRNA–target interactions database (cuhk.edu.cn)), and miRDB (miRDB—microRNA target prediction database). Target genes existing across all three databases were deemed as acceptable. As previously mentioned, GO and KEGG analyses of these predicted target genes were conducted. The network among the pyroptosis-related DElncRNAs, pyroptosis-related DEGs, and their mutual target miRNAs was analyzed in Cytoscape 3.9.1. The clusters isolated from the network were analyzed in MCODE (Version 2.0.3), and the hub genes of the network were discovered using CytoHubba (Version 0.1) [83,84,85].

### 2.11. Statistical Analyses

The correlation between the risk scores and the tumor-infiltrating immune cell proportion was calculated through performing Spearman’s correlation analysis. The Kruskal–Wallis test compared the tumor-infiltrating immune cell proportions among the clusters assessed. The Wilcox test compared the immune checkpoint gene expressions of the high- and low-risk groups, and those of the subtypes were compared using the Kruskal–Wallis test. In the current work, statistical analyses were conducted in R. 4.3.0 with a significant threshold of *p* < 0.05.

## 3. Results

### 3.1. The Establishment and Verification of the Prognostic Pyroptosis-Related LncRNA Signature

#### 3.1.1. The Identification and Functional Analysis of the Pyroptosis-Related DEGs

**Figure 1:**
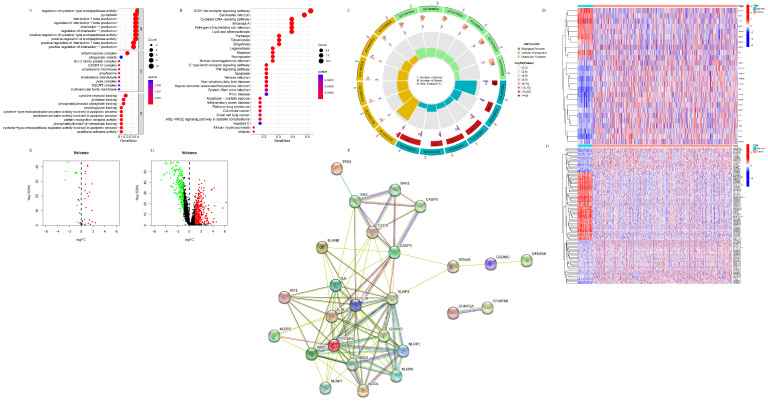
Functional analysis of the pyroptosis-related DEGs. (**A**) Bubble plot of GO analysis of the 26 pyroptosis-related DEGs. (**B**) Bubble plot of KEGG analysis of the 26 pyroptosis-related DEGs. (**C**) Circle plot of GO analysis of the 26 pyroptosis-related DEGs. (**D**) Heatmap of the 26 pyroptosis-related DEGs. (**E**) Volcano plot of the 26 pyroptosis-related DEGs. (**F**) Protein–protein interaction of the 26 pyroptosis-related DEG. (**G**) Volcano plot of the 543 pyroptosis-related DElncRNAs. (**H**) Heatmap of the 543 pyroptosis-related DElncRNAs. In (**E**,**G**), red color refers to up-regulation, green color refers to down-regulation, black color refers to changes with no statistical significance.

In total, twenty-six pyroptosis-related DEGs (BAK1, BAX, CASP3, CASP6, CHMP2A, CHMP4B, CYCS, ELANE, CSDMC, GSDMD, GSDME, IL18, IL-1B, IRF1, NLRP7, NLRP6, NLRP3, NLRP2, NLRP1, NOD2, NOD1, PJVK, PYCHARD, TP63, AIM2, and IL6) were identified, in which nine (TP63, IL6, ELANE, NLRP1, PJVK, GSDME, NOD1, NLRP3, and IL1B) were downregulated in the tumor samples, while seventeen (CHMP2A, CYCS, CHMP4B, CASP3, CASP6, IRF1, GSDMD, BAK1, BAX, NLRP2, IL18, NLRP6, NOD2, PYCARD, AIM2, GSDMC, and NLRP7) were upregulated, respectively. The GO and KEGG analysis of the 26 pyroptosis-related DEGs are shown in Figure 1A–C. In GO analysis, the 26 pyroptosis-related DEGs were primarily enriched in the pyroptosis item itself and their function items were determined to be related to IL-1, the cysteine-type endopeptidase, and the inflammasome complex, which are parts of pyroptosis. In KEGG analysis, they were primarily enriched in the NOD-like receptor signaling pathway and the cytosolic DNA-sensing pathway, which are also parts of pyroptosis, in addition to various infectious diseases, cancer diseases, and inflammatory diseases, indicating the essential role exerted by pyroptosis in these physiological and pathological processes. Interestingly, these pyroptosis-related DEGs were also enriched in apoptosis and necroptosis, with the former, according to the GO analysis result, indicating the intrinsic correlation between pyroptosis, apoptosis, and necroptosis. The heatmap and volcano of the 26 pyroptosis-related DEGs are shown in Figure 1D,E. The results of the protein–protein interaction analysis is shown in Figure 1F. The volcano of the 543 pyroptosis-related DElncRNAs is shown in Figure 1G. From the whole level, the differences in the pyroptosis-related DEGs between the normal and the tumor tissues were quite clear. In contrast, the heterogenicity of the pyroptosis-related DEGs within the tumor tissues was higher than those in the normal tissues, which is in accordance with the heterogenicity of breast cancer and provided theoretical support for the further classification of its subtypes. According to the protein–protein interactions, BAX, BAK1, and CYCS are essential parts of both apoptosis and pyroptosis and are also closely related to CASP3, indicating that apoptosis is probably related to pyroptosis through the caspase-3/GSDME pathway. Meanwhile, PYCARD promotes apoptosis through caspase-8/9 and is also closely related to the sensor (NLRP1/2/3/6/7, AIM2, and NOD1/2), executor (GSDMD), and inflammatory (IL-18, IL-1β) factors of pyroptosis, indicating that apoptosis could also be related to pyroptosis through the canonical pathway.

#### 3.1.2. The Identification of the Pyroptosis-Related DElncRNAs

In total, 2279 lncRNAs were identified as correlated with 52 pyroptosis-related genes, and 543 were differentially expressed between the tumor and the normal samples, respectively. The volcano and heatmap of the pyroptosis-related DelncRNAs are shown in Figure 1G,H. Similarly, the differences in the pyroptosis-related DElncRNAs between the normal and tumor tissues were quite evident from the whole level. In contrast, the heterogenicity of the pyroptosis-related DElncRNAs within the tumor tissues was higher than those of the normal tissues, which further verified the heterogenicity of breast cancer and provided more theoretical support for the further classification of its subtypes.

#### 3.1.3. The Establishment of the Prognostic Pyroptosis-Related LncRNA Signature

**Figure 2:**
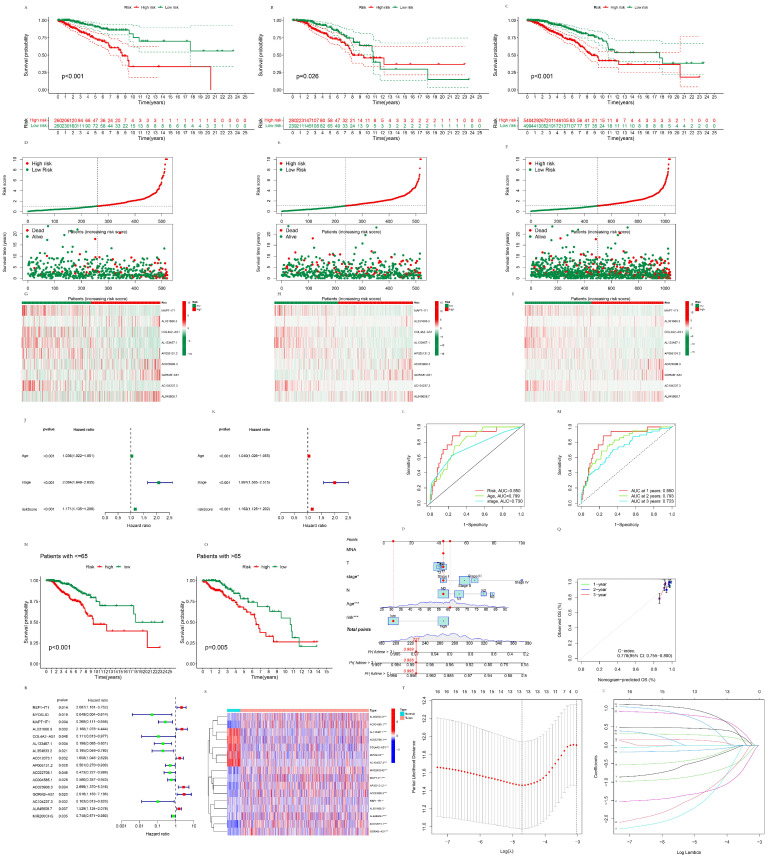
The validation of the prognostic pyroptosis-related lncRNA signature. (**A**) K–M curves of the high-risk group and low-risk group in the train dataset. (**B**) K–M curves of the high-risk group and low-risk group in the test dataset. (**C**) K–M curves of the high-risk group and low-risk group in the whole dataset. (**D**) Risk plot of the high-risk group and low-risk group in the train dataset. (**E**) Risk plot of the high-risk group and low-risk group in the test dataset. (**F**) Risk plot of the high-risk group and low-risk group in the whole dataset. (**G**) Heatmap of the signature lncRNAs of the high-risk group and low-risk group in the train dataset. (**H**) Heatmap of the signature lncRNAs of the high-risk group and low-risk group in the test dataset. (**I**) Heatmap of the signature lncRNAs of the high-risk group and low-risk group in the whole dataset. (**J**) Univariate Cox proportional hazard regression analysis of the risk score, age and stage. (**K**) Multivariate Cox proportional hazard regression analysis of the risk score, age and stage. (**L**) ROC curves of the risk score, age and stage at 1st year. (**M**) ROC curves of the risk score at 1st year, 2 years, and 3 years. (**N**) K–M curves of the high-risk group and low-risk group patients aged ≤ 65. (**O**) K–M curves of the high-risk group and low-risk group patients aged > 65. (**P**) Nomogram of the pyroptosis-related LncRNA signature. * *p* < 0.05; ** *p* < 0.01; *** *p* < 0.001 (**Q**) The associated calibration curve of the nomogram. (**R**) Significant pyroptosis-related lncRNA in univariate Cox analysis. (**S**) Heatmap of the expressions of the significant pyroptosis-related lncRNAs in univariate Cox analysis between the normal and tumor groups. (**T**) Minimum lambda value of Lasso regression. (**U**) A coefficient profile plot of Lasso regression.

Patients were partitioned randomly into the training group and the test group, respectively. Univariate Cox analysis of OS was conducted with the pyroptosis-related DElncRNAs, and 16 of them (RSF1-IT1, MYOSLID, MAPT-IT1, AL031666.3, COL4A2-AS1, AL133467.1, AL354833.2, AC012073.1, AP005131.2, AC022706.1, AC004585.1, AC023908.3, GORAB-AS1, AC104237.3, AL645608.7, and MIR200CHG) were found to be statistically significant (Figure 2A,B). These significant lncRNAs in univariate Cox analysis were subsequently subjected to Lasso regression, and 13 lncRNAs (RSF1-IT1, MAPT-IT1, AL031666.3, COL4A2-AS1, AL133467.1, AL354833.2, AC012073.1, AP005131.2, AC023908.3, GORAB-AS1, AC104237.3, AL645608.7, and MIR200CHG) were found to be statistically significant (Figure 2C,D). These significant lncRNAs in Lasso regression were then subjected to multivariate Cox analysis, and the prognostic model was ultimately described as follows:

Risk score = (−1.1464 × expression MAPT-IT1) + (1.5165 × expression AL031666.3) + (−2.2865 × expression COL4A2-AS1) + (−1.8048 × expression AL133467.1) + (−0.5627 × expression AP005131.2) + (0.8395 × expression AC023908.3) + (1.3219 × expression GORAB-AS1) + (−2.0471 × expression AC104237.3) + (0.3279 × expression AL645608.7).

#### 3.1.4. The Validation of the Prognostic Pyroptosis-Related LncRNA Signature

K–M curves were drawn to validate the prognostic value of the pyroptosis-related lncRNA signature on the OS, which was significantly lower in the high-risk group compared to the low-risk group in the training dataset (*p* < 0.001), test dataset (*p* = 0.026), and whole dataset (*p* < 0.001) (Figure 2A–C), indicating that the signature was of prognostic value in predicting the survival of BC patients. The risk plots of patients in the high- and low-risk groups in the training, test, and whole datasets were also drawn, and it was found that mortality was positively correlated with the risk scores (Figure 2D–F). The heatmap of lncRNA expression of the prognostic model in the high- and low-risk groups in the train dataset, test dataset, and whole dataset were also shown in Figure 2G–I. The risk score was of significant prognostic value in both the univariate (*p* < 0.001, hazard ratio 1.171) and multivariate Cox proportional hazard regression models (*p* < 0.001, hazard ratio 1.162), as shown in Figure 2J,K. ROC curves were drawn to determine the specificity and sensitivity of the lncRNA signature in predicting the prognosis of BC patients. The AUC of the LncRNA signature was 0.850, which was higher than the 0.799 value obtained with age and the 0.730 calculated regarding the stage in the 1st year (Figure 2L), indicating the better prognostic potential of the signature than age and the stage. The AUC at one year, two years, and three years were 0.850, 0.793, and 0.723, respectively, which indicated that the prognostic potential of the signature showed a higher accuracy in the 1st year and was also acceptable in two years and three years (Figure 2M). The patients were partitioned into two groups based on their age. K–M curves were drawn to evaluate the prognostic value of the pyroptosis-related lncRNA signature on the OS of patients ≤65 and >65, respectively. It was shown that for patients ≤65 (*p* < 0.001) and >65 (*p* = 0.005), the OS was significantly higher in the low-risk group than the high-risk group (Figure 2N,O). The nomogram of this pyroptosis-related lncRNA signature and their associated calibration curve are shown in Figure 2P,Q.

### 3.2. The Functional Analysis of the Pyroptosis-Related LncRNA Signature

#### 3.2.1. Differentially Expressed Genes between the High- and Low-Risk Groups

**Figure 3:**
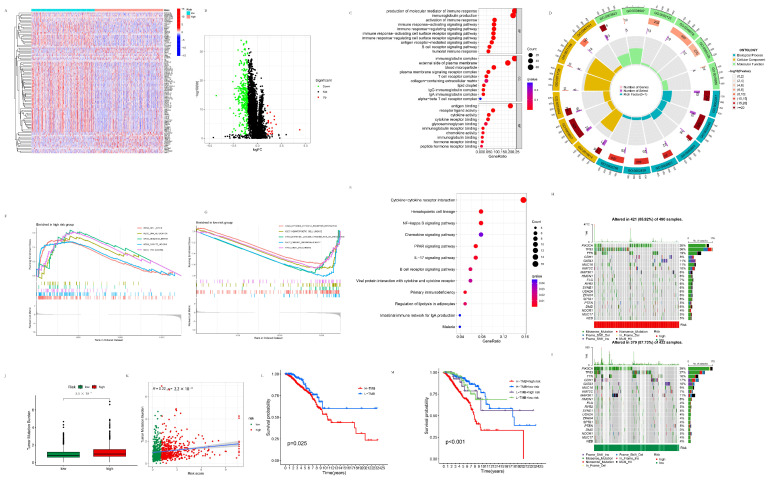
Functional analysis of the risk-related DEGs. (**A**) Heatmap of 350 risk-related DEGs. (**B**) Volcano plot of 350 risk-related DEGs. (**C**) Bubble plot of GO analysis of 350 risk-related DEGs. (**D**) Circle plot of GO analysis of 350 risk-related DEGs. (**E**) Bubble plot of KEGG analysis of 350 risk-related DEGs. (**F**) KEGG pathways of the cell cycle, DNA replication, mismatch repair, oocyte meiosis, and proteasome enriched in the high-risk group. (**G**) KEGG pathways of the cytokine–cytokine receptor interaction, hematopoietic cell lineage, the intestinal immune network for IGA production, primary immunodeficiency, and viral myocarditis enriched in the low-risk group. (**H**) TMB analysis of the high-risk group. (**I**) TMB analysis of the low-risk group. (**J**) Boxplot of TMB in the high- and low-risk groups. (**K**) Correlation between the TMB and the risk score. (**L**) K–M curves of the high- and low-risk TMB patients. (**M**) K–M curves of the high risk + high TMB, high risk + low TMB, low risk + high TMB, and low risk + low TMB groups.

In total, 350 DEGs between the high- and low-risk groups were identified, as shown in Figure 3A,B. According to the heatmap and volcano map, DEGs downregulated in the high-risk group compared with the low-risk group were more than those that were upregulated, potentially indicating that the worsened prognosis of the high-risk group could be attributed more to the suppression of genes with cancer-inhibition activity. According to KEGG and GO analysis, these risk-related genes were primarily enriched in “production of molecular mediator of immune response” and “immunoglobulin production” in the biology process (BP), immunoglobulin complex in CCs (cellular components) and antigen binding in the MF (molecular function) of GO as, shown in Figure 3C,D, and “Cytokine-cytokine receptor interaction” of KEGG, as shown in Figure 3E, respectively. It is indicated that the 350 DEGs between the high- and low-risk groups were primarily associated with the immune reaction, especially the antibody production, antigen–antibody reaction, and cytokine–cytokine interaction parts, which potentially further influenced the innate immunity to cancer along with the remodeling of the tumor immune microenvironment (TIME) in BC and correspondingly to the prognosis of BC patients.

#### 3.2.2. GSEA Analysis of the High- and Low-Risk Groups

As shown in Figure 3F,G, the KEGG pathways of the cell cycle, DNA replication, mismatch repair, oocyte meiosis, and proteasome were enriched in the high-risk group, while the KEGG pathways of the cytokine–cytokine receptor interaction, hematopoietic cell lineage, intestinal immune network for IGA production, primary immunodeficiency, and viral myocarditis were enriched in the low-risk group, respectively. This result indicated that tumor cells were possibly under active proliferation in the high-risk group, and their associated pathways, such as cell cycle, DNA replication, and mismatch repair, were all upregulated as a result. On the other hand, the low-risk group was in a relatively “cool” state of inflammation. Thus their corresponding pathways, like cytokine–cytokine receptor interaction, hematopoietic cell lineage, and the intestinal immune network for IGA production, were all downregulated. In previous research, it was widely discussed in that pyroptosis typically exhibits the benefit of directly killing tumor cells, but under an inflammatory environment is advantageous for tumor migration and metastasis. Our results also supported this view.

#### 3.2.3. TMB Analysis of the High- and Low-Risk Groups

As shown in Figure 3H,I, the proportions of mutated PIK3CA, TP53, and CDH1 in the high-risk group were 30%, 36%, and 8%, in comparison with that of 39%, 27%, and 17 in the low-risk group, respectively. As shown in Figure 3J,K, TMB was significantly higher in the high-risk group than in the low-risk group (*p* = 5.5 × 10^−7^), and TMB was positively correlated with the risk scores (R = 0.22, *p* = 2.2 × 10^−11^). K–M curves were drawn to determine the prognostic potential of the TMB and the TMB + risk score on the overall survival of patients. The overall survival of the high TMB group was significantly lower than that of the low TMB group (*p* = 0.025). The overall survival of the high TMB + high-risk group was significantly different from that of the high TMB + low-risk group (*p* = 6.7 × 10^−9^), low TMB + high-risk group (*p* = 0.018), and low TMB + low-risk group (*p* = 0.001) (Figure 3L,M).

#### 3.2.4. TME Analysis of the High- and Low-Risk Groups

**Figure 4:**
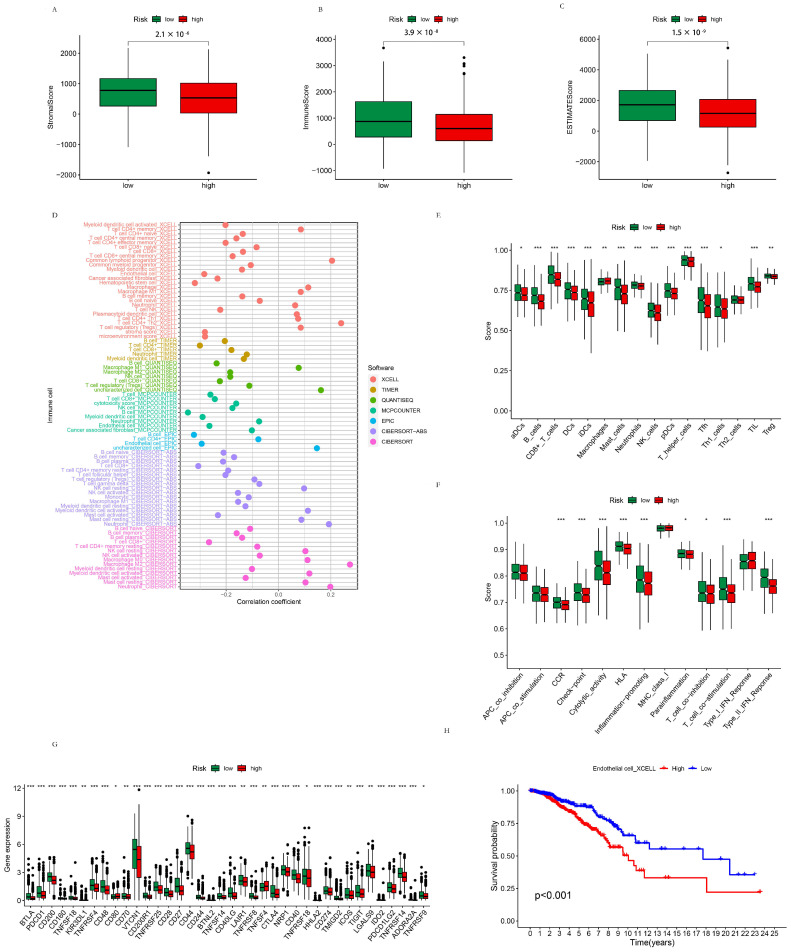
TME analysis of the high- and low-risk groups. (**A**) The stromal scores of the high-risk group and the low-risk group. (**B**) The immune score of the high-risk group and the low-risk group. (**C**) The estimate score of the high-risk group and the low-risk group. (**D**) The correlation between the immune cell proportion and their risk score. (**E**) The immune cell scores of the high- and low-risk groups. (**F**) The immune function scores of the high- and low-risk groups. (**G**) The expression of the checkpoint genes of the high- and low-risk groups. (**H**) Drug sensitivity of the high- and low-risk groups to Aliseritib. * *p* < 0.05; ** *p* < 0.01; *** *p* < 0.001.

The stromal score (*p* = 2.1 × 10^−6^), immune score (*p* = 3.9 × 10^−8^) and estimate score (*p* = 1.5 × 10^−9^) were all significantly lower in the high-risk group than in the low-risk group, respectively (Figure 4A–C). This result is in accordance with our previous analyses of GO, KEGG, and GSEA of the DEGs between the high-risk group and low-risk group, and further strengthened the reliability of the assumption in that the difference between the prognosis of the high-risk group and low-risk group was probably attributed to the DEGs that regulated the immune reaction and remodeled the TME. The correlation between the immune cell proportion and the risk score is displayed in Figure 4D. According to the results of most of the software used in this study, the T cell CD4+, T cell CD8+, B cell, neutrophil, macrophage M2, NK cell, endothelial cell, and myeloid dendritic cell populations exhibited relatively higher correlations with the risk score. In most of the results obtained from the seven software tools implemented in this study, the activated myeloid dendritic cells, CD4+ T cells, CD8+ T cells, B cells, Tregs, active NK cells, active mast cells, macrophage M2, and endothelial cell populations were negatively correlated with the risk score. Meanwhile, resting NK cells, resting mast cells, macrophage M1, resting myeloid dendritic cells, and neutrophils were positively correlated with the risk score, indicating that having an effectively activated immune system to combat tumors was advantageous to prognosis of BC. In this case, neutrophils were mostly positively correlated with their risk score, which was possible due to their tumor-promoting functions, such as supporting angiogenesis and metastasis, instead of its anti-tumor activity, including phagocytosis, release of cytotoxic granules, and generation of neutrophil extracellular traps (NETs). The K–M analyses of the overall survival and proportion of endothelial cells calculated using XCELL software are shown in Figure 4E. The K–M analyses of the overall survival and proportion of multiple immune cells calculated using various software are displayed in Figure S1. The contents of B cell plasma, B cell, cancer-associated fibroblast, class-switched memory B cell, endothelial cell, hematopoietic cell, macrophage M0/1/2, activated NK cell, CD8+ T cell, T cell follicular helper, and Treg populations were significantly correlated with the survival possibility of patients, indicating that the above-mentioned immune cells exerted pivotal roles in TME remodeling and in the prognosis of BC. ssGSEA was performed using the immune.gmt dataset. The immune cell and function scores were compared between the high- and low-risk groups (Figure 4F,G). As for immune cell scores, aDCs, Th1_cells (*p* < 0.05); macrophages, Treg (*p* < 0.01); CD8+_T_cells, neutrophils, iDCs, Mast_cells, NK_cells, B_cells, DCs, Tfh, and TIL (*p* < 0.001) were all found to be significantly lower in the high-risk group compared to the low-risk group. Macrophages were also significantly higher in the high-risk group than the low-risk group. As for the immune function scores, Parainflammation, T_cell_co-inhibition (*p* < 0.05); T_cell_co-stimulation, Cytolytic_activity, Type_II_IFN_Response, CCR, inflammation-promoting, Checkpoint, andHLA (*p* < 0.001) were significantly lower in the high-risk group compared to the low-risk group. The expression of the checkpoint genes of the high- and low-risk groups are shown in Figure 4H. CD80, TNFRSF18, TNFRSF9 (*p* < 0.05); KIR3DL1, CD70, LAIR1, TNFSF4, ICOS, LGALS9 (*p* < 0.01); BTLA, PDCD1, CD200, CD160, TNFSF18, TNFRSF4, CD48, VTCN1, CD200R1, TNFRSF25, CD28, CD27, CD44, CD244, BTNL2, TNFSF14, CD40LG, TNFRSF8, CTLA4, NRP1, CD40, HHLA2, CD274, TMIGD2, TIGIT, IDO2, PDCD1LG2, TNFRSF14, and ADORA2A (*p* < 0.001) were all differentially expressed between the high- and low-risk groups with statistical significance. As an example, the drug sensitivity of the high- and low-risk groups to Aliseritib was predicted using the machine learning method and is shown in Figure 4I. Drug sensitivity of the high- and low-risk groups to parts of drugs is shown in Figure S2.

### 3.3. The Subtypes of Breast Cancer Patients Based on Their Risk Score

#### 3.3.1. The Clustering of Breast Cancer Patients Using the Consensus Cluster Method

**Figure 5:**
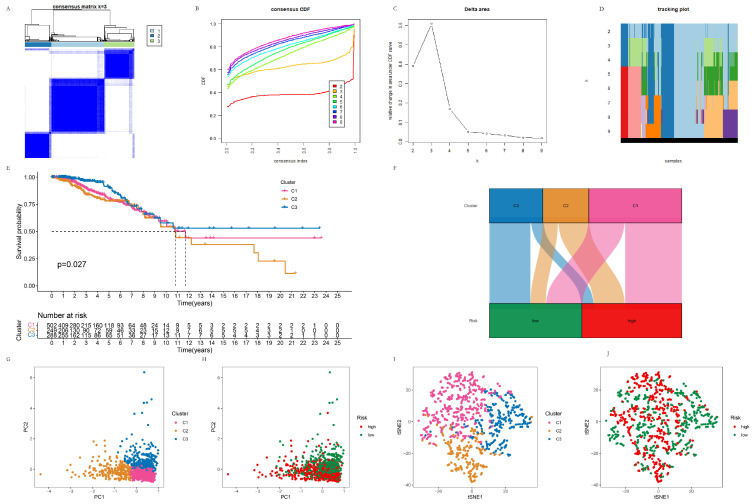
The subtypes of breast cancer patients are classified based on the risk score. (**A**) Clustering BC patients into three subtypes. (**B**) Consensus CDF of the clustering. (**C**) Delta area of the clustering. (**D**) Tracking plot of the clustering. (**E**) K–M curves of C1, C2, and C3 clusters. (**F**) The distribution of the C1, C2, and C3 patients in the high- and low-risk groups. (**G**) The PCA analysis of the C1, C2 and C3 clusters. (**H**) The PCA analysis of the high- and low-risk groups. (**I**) The t-SNE analysis of the C1, C2, and C3 clusters. (**J**) The t-SNE analysis of the high- and low-risk groups.

Breast cancer patients were clustered using the R package “ConsensusClusterPlus” using partitioning around medoids for clustering and the Euclidean distance for calculating the distance between data points, as shown in Figure 5A–D. Kaplan–Meier curves were drawn to evaluate the prognostic value of the risk-based subtype on the OS, which was significantly higher in the C2 than C3 cluster (*p* = 0.027) (Figure 5E). The distribution of the C1, C2, and C3 patients in the high- and low-risk groups is shown in Figure 5F. The PCA analysis and t-SNE analysis were performed on the high- and low-risk groups, as well as on the C1, C2, and C3 clusters; the results are shown in Figure 5G–J. The PCA and t-SNE analyses separated the C1, C2, and C3 clusters. At the same time, the high- and low-risk groups were better separated wit the PCA analysis than by the t-SNE analysis.

#### 3.3.2. TME Analysis of the C1, C2, and C3 Clusters

**Figure 6:**
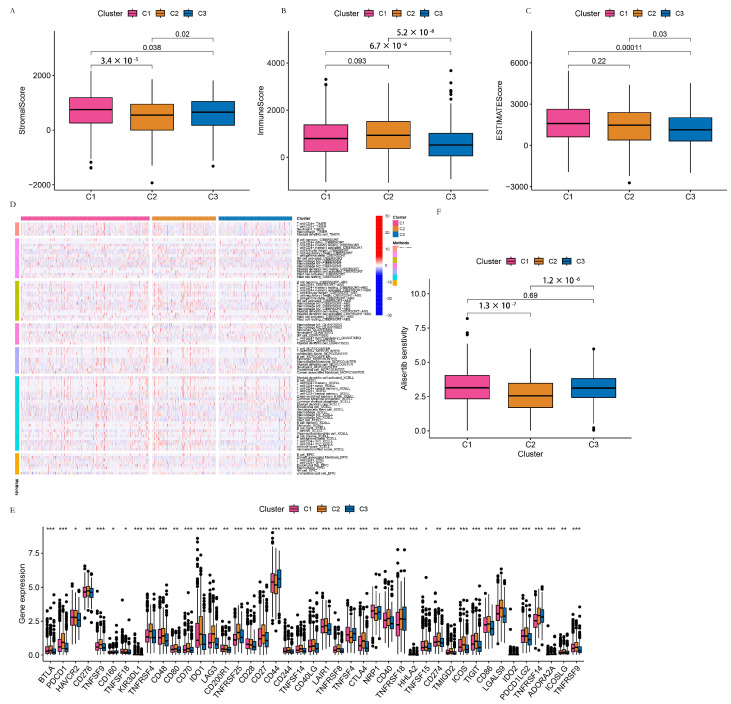
TME analysis of the C1, C2, and C3 clusters. (**A**) The stromal score of the C1, C2, and C3 clusters. (**B**) The immune score of the C1, C2, and C3 clusters. (**C**)The estimate score of the C1, C2 and C3 clusters. (**D**) The correlation between the immune cell proportion and their risk score. (**E**) The expression of the checkpoint genes of the C1, C2 and C3 clusters. (**F**) Drug sensitivity of the C1, C2 and C3 clusters to Aliseritib. * *p* < 0.05; ** *p* < 0.01; *** *p* < 0.001.

The stromal score of the C1 cluster was significantly higher than that of the C2 (*p* = 3.4 × 10^−5^) and C3 (*p* = 0.038) clusters, respectively, and the stromal score of the C3 cluster was significantly higher than that of C2 cluster (*p* = 0.02). The immune scores of the C1 (*p* = 6.7 × 10^−6^) and C2 (*p* = 5.2 × 10^−8^) clusters were significantly higher than that of the C3 cluster. The estimate scores of the C1 (*p* = 0.00011) and C2 (*p* = 0.03) clusters were significantly higher than that of the C3 cluster, as shown in Figure 6A–C. The immune infiltration of the C1, C2, and C3 clusters was compared using seven software tools. The heatmap is shown in Figure 6D. The expression of the checkpoint genes of the C1, C2, and C3 clusters is shown in Figure 6E. HAVCR2, CD160, TNFSF18, TNFSF15 (*p* < 0.05); CD276, CD80, CD200R1, NRP1, CD274, ICOSLG (*p* < 0.01); BTLA, PDCD1, TNFSF9, KIR3DL1, TNFRSF4, CD48, CD70, IDO01, LAG3, TNFRSF25, CD28, CD27, CD44, CD244, TNFSF14, CD40LG, LAIR1, TNFRSF8, TNFSF4, CTLA4, CD40, TNFRSF18, HHLA2, TMIGD2, ICOS, TIGIT, CD86, LGALS9, IDO2, PDCD1LG2, TNFRSF14, ADORA2A, and TNFRSF9 (*p* < 0.001) were all differentially expressed among the C1, C2 and C3 clusters with statistical significance. The drug sensitivity of the C1, C2, and C3 clusters was predicted using the machine learning method, and the sensitivity to Alisertib as an example is shown in Figure 6F. Drug sensitivity to parts of drugs is shown in Figure S3.

### 3.4. The Construction and Analysis of the Pyroptosis-Related ceRNA Regulatory Network

#### 3.4.1. The Establishment of the Pyroptosis-Related ceRNA Regulatory Network

**Figure 7:**
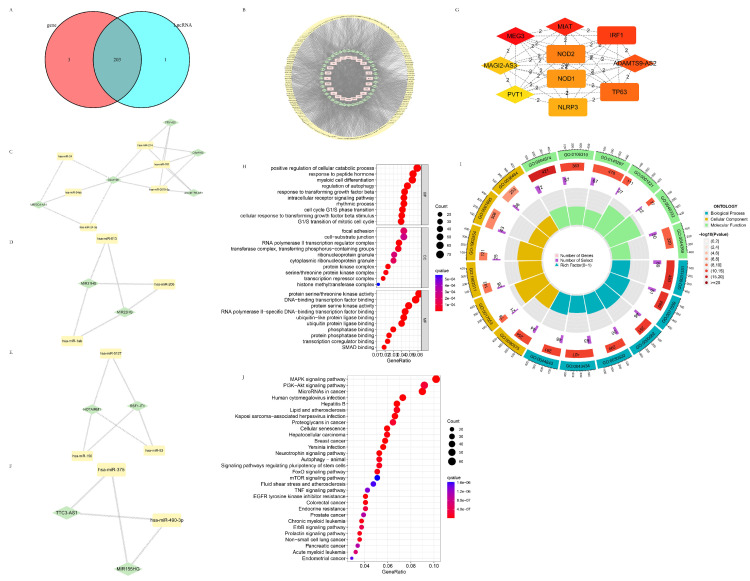
The construction and analysis of the pyroptosis-related ceRNA regulatory network. (**A**) Intersection of miRNA predicted by the pyroptosis-related DEGs and the DElncRNAs. (**B**) Pyroptosis-related ceRNA regulatory network constructed by the pyroptosis-related DEGs, DElncRNAs, and the intersection of the predicted miRNA. (**C**) Cluster 1 (score 3.6) of the pyroptosis-related ceRNA regulatory network. (**D**) Cluster 2 (score 3.0) of the pyroptosis-related ceRNA regulatory network. (**E**) Cluster 3 (score 3.0) of the pyroptosis-related ceRNA regulatory network. (**F**) Cluster 4 (score 2.667) of the pyroptosis-related ceRNA regulatory network. (**G**) The hub genes with the shortest paths obtained from the pyroptosis-related ceRNA regulatory network through CytoHubba. (**H**) Bubble plot of GO analysis of the potential target genes predicted using the intersection miRNA. (**I**) Circle plot of GO analysis of the potential target genes predicted using the intersection miRNA. (**J**) Bubble plot of KEGG analysis of the potential target genes predicted using the intersection miRNA.

Pyroptosis-related DEGs, DElncRNAs, and the intersection of miRNA predicted by the above two (Figure 7A) were used to establish the pyroptosis-related ceRNA regulatory network (Figure 7B). Cluster 1 (Score 3.6), Cluster 2 (Score 3.0), Cluster 3 (Score 3.0), and Cluster 4 (Score 2.667), which were obtained from the pyroptosis-related ceRNA regulatory network through MCODE, are shown in Figure 7C–F. The hub genes with the shortest paths obtained from the pyroptosis-related ceRNA regulatory network through CytoHubba are shown in Figure 7G. The darker color the node, the more critical it is in the pyroptosis-related ceRNA network, as predicted with the MCC algorithm. It was indicated that MEG3, MIAT, IRF1, ADAMTS9-AS2, and TP63 are among the essential factors in this network.

#### 3.4.2. Potential Target Genes of the Intersection Target miRNA Predicted by the Pyroptosis-Related DEGs and DELncRNAs

The potential target genes of the intersection target miRNA were searched in three databases, including TargetScan (TargetScanHuman 7.1), miRTarBase(miRTarBase: the experimentally validated microRNA–target interactions database (cuhk.edu.cn)), and miRDB (miRDB—microRNA target prediction database), and candidates existing in all the three databases were taken as acceptable. Pyroptosis-related DEGs shared almost all the predicted miRNAs (203/206) with the pyroptosis-related DElncRNAs (203/204), indicative of this prediction’s high reliability. The GO and KEGG analyses are shown in Figure 7H–J. The genes were mainly enriched in the “positive regulation of cellular catabolic process” and “response to peptide hormone” in BP, “focal adhesion” and “cell-substrate junction” in CC, and “protein serine/threonine kinase activity” and “protein-binding transcription factor binding” in MF, accompanied with various gene ontologies related to protein kinase, the cell cycle, and transcription. The genes mainly were enriched in the “PI3K-Akt signaling pathway”, “microRNAs in cancer”, and the “MAPK signaling pathway” pathways in KEGG, as well as multiple cancer, infectious diseases, and autophagy. On the one hand, these miRNAs regulated pyroptosis-related processes through pyroptosis-related DElncRNAs and DEGs. On the other hand, these miRNAs participated in autophagy, multiple cancer species, various well-known tumor-associated processes, and pathways, such as the cell cycle, cell adhesion, the MAPK pathway, the PI3K-AKT pathway, the FoxO pathway, and the mTOR pathway through other target genes. These miRNAs might represent the switch between pyroptosis and autophagy, and provide a new connection between pyroptosis, cancer, and cancer-related signaling pathways.

## 4. Discussion

Our study focused on constructing a pyroptosis-related, long non-coding RNA (lncRNA) signature for the prognosis of BC and for the comprehensive analysis of the tumor microenvironment (TME) associated with the risk scores. We also identified three breast cancer subtypes based on the risk scores obtained and explored the TME associated with the three subtypes. We derived a nine lncRNA prognostic signature (RSF1-IT1, MAPT-IT1, AL031666.3, COL4A2-AS1, AL133467.1, AL354833.2, AP005131.2, AC023908.3, GORAB-AS1, AC104237.3, AL645608.7, and MIR200CHG), which significantly stratified patients into high- and low-risk groups with distinct OS outcomes. Our findings highlighted the potential clinical applicability of these lncRNAs as biomarkers for personalized prognostication and for potential therapeutics targeting breast cancer.

Pyroptosis, a highly inflammatory form of programmed cell death, was implicated in various malignancies, including breast cancer [86]. The potential of pyroptosis as a novel cancer therapeutic strategy has been previously recognized [87], and our findings further emphasized the necessity of understanding the mechanisms of pyroptosis in breast cancer. Among the twenty-six pyroptosis-related DEGs identified in breast cancer, nine were downregulated, and seven were upregulated, respectively. AIM2, a double-strand DNA sensor in the inflammasome of pyroptosis, was also found to be upregulated in the tumor samples of this work, which indicated active pyroptosis in BC tissue [88]. IL-18 and GSDMD, both constituents of the canonical pathway of pyroptosis, were also upregulated in the tumor samples that were assessed, further proving the activation stage of pyroptosis in BC. ELANE (elastase, neutrophil expressed), which regulates the functions of NK cells, monocytes, and granulocytes, could kill tumor cells and suppress tumorigenesis [89]. The significant downregulation of ELANE observed in the tumor samples assessed was probably related to the inhibited tumor immunity in the BC patients, which is in accordance with a previous study [89], and with our analysis of the DEGs between the high-risk and low-risk groups.

Our study explored the potential role of lncRNAs in regulating pyroptosis in BC. In total, 2279 lncRNAs were considered to be related to pyroptosis, and 543 of these were identified as DElncRNAs. Among these DElncRNAs, some were proven to participate in cancer development, invasions, progressions, and drug resistance, such as MEG3 [41,48], MIAT [46], and MIR200CHG [90]. The comprehensive identification of these lncRNAs may expand the repertoire of molecules implicated in pyroptosis and highlight potential targets for therapeutic intervention. We identified and validated a lncRNA signature with powerful prognostic value for breast cancer in silico, providing a valuable prognostic tool and potential therapeutic targets. These lncRNAs could potentially influence breast cancer progression by regulating pyroptosis, but further investigations are needed to uncover their exact molecular mechanisms.

Furthermore, according to our findings, the lncRNA signature theoretically demonstrated a higher accuracy compared to the conventional clinical characteristics, such as age and stage, in predicting the prognosis of BC patients and revealed a promising prognostic potential. We used stringent statistical methods, including univariate Cox, Lasso, and multivariate Cox analyses, to select the most significant lncRNAs. This robust selection process increased the credibility and validity of our signature. This signature exhibited a good predictive power for patient survival, with an AUC of 0.850 in the training dataset and 0.793 in the test dataset, respectively, and was determined to be superior compared to the conventional clinical variables like age and stage. Remarkably, it maintained its predictive value across different age groups, reinforcing its general applicability.

The interrelationship between the TMB and the lncRNA signature was another crucial finding. The higher TMB in the high-risk group and its positive correlation with the risk score suggested a possible linkage between genomic instability and the risk associated with the lncRNA signature, echoing the increasing recognition of the TMB as a prognostic biomarker in cancer [91,92]. The Kaplan–Meier analyses further strengthened this association, emphasizing the potential of integrating the TMB and the lncRNA signature for a better prognostic accuracy. Our signature might therefore guide personalized immunotherapeutic decisions in the future.

Significantly, our study explored the interplay between lncRNAs, pyroptosis, and the TME. The differential gene expression observed between the high- and low-risk groups suggested an active interplay between the pyroptosis-related lncRNA signature and the immune response. The enriched pathways observed in the GO and KEGG analysis, including the “production of molecular mediator of an immune response”, “immunoglobulin production”, and “Cytokine-cytokine receptor interaction”, supported the role of the signature in modulating the immune response in the context of breast cancer. This enrichment resonates with recent studies illustrating the immunomodulatory potential of lncRNAs [93,94]. GSEA analysis showed that distinct KEGG pathways were enriched in both the high- and low-risk groups, further indicating the multi-faceted role of the lncRNA signature. Notably, cell cycle-related pathways were more enriched in the high-risk group, aligning with the previous literature highlighting the importance of cell cycle dysregulation observed in aggressive breast cancer cell subtypes [95].

TME analysis brought forth the complex immunologic landscape associated with the lncRNA signature. Reduced stromal and immune scores in the high-risk group denoted a more immunosuppressive environment, aligning with the widely accepted notion of an immunosuppressive TME contributing to cancer progression [96]. The varying associations between the different immune cell types and risk scores underlined the intricate interplay between the various components of the immune response and the signature. Observing a higher correlation of CD4+ T cells, CD8+ T cells, B cells, neutrophils, macrophage M2, NK cells, endothelial cells, and myeloid dendritic cells with the risk scores is intriguing. These immune cells have been widely reported to play varied roles in tumorigenesis, depending on their activation state and their surrounding milieu [97]. This highlighted the complexity of the immune landscape in the context of breast cancer risk and pyroptosis, requiring a more nuanced exploration.

The differential expression of immune checkpoint genes, another hallmark of immune evasion in cancer, further stressed the potential role of the lncRNA signature in immune modulation [98]. Our immune checkpoint gene expression analysis revealed a differential expression between the high- and low-risk groups (Figure 4H). Immune checkpoints, such as PD1/PDL1 and CTLA4, are critical regulators of the immune response, and their overexpression in tumor cells is a well-known mechanism of immune evasion [99]. Therapeutic blockade of these checkpoints has shown a considerable level of success in various cancers, suggesting that our high-risk group may benefit from these therapies. The expression of checkpoint proteins was almost all higher in the low-risk group than the high-risk group with the exception of CD80, which possibly explained the different outcomes of these two groups of patients and indicated that blocking CD80 would benefit the high-risk patients more than the low-risk patients.

These results might further illuminate the intricate links between the lncRNAs, pyroptosis, and TME remodeling in breast cancer, which is crucial for optimizing immunotherapeutic strategies. Lastly, the prediction of differential drug sensitivity between the high- and low-risk groups opens up possibilities for personalized treatment strategies. Though preliminary, this finding could emphasize the potential utility of the lncRNA signature in guiding therapeutic decision making.

Based on the risk scores obtained, we performed consensus clustering using partitioning around medoids and Euclidean distance calculations and identified three distinct clusters labeled C1, C2, and C3, respectively. K–M curves showed that the OS of the C2 cluster was significantly higher than that of the C3 cluster, which suggested that the risk-based subtypes had varying prognostic values. However, no significant differences were observed between the C1 and C2 and C1 and C3 clusters. The clustering indicated heterogeneity within breast cancer that could be attributed to the differential activation of pyroptosis and consequent variations in the TME. This reinforced the need for personalized treatments tailored to the individual tumor characteristics.

t-SNE analysis and principal component analysis (PCA) results showed that C1, C2, and C3 clusters were well separated, while the high- and low-risk groups were not as distinctly distinguishable. The unclear separation of the high- and low-risk groups could suggest a complex biological overlap despite the risk status, an aspect that deserves further investigation.

In the TME analysis of the C1, C2, and C3 clusters, the C1 cluster showed significantly higher estimate, immune, and stromal scores than the other clusters. This might suggest an active TME in the C1 cluster, potentially enhancing immune surveillance and thus resulting in a better prognosis. The distributions of the positive and negative z-scores of immune cell species were markedly different between the clusters, indicating variations in immune infiltration between subtypes.

The expression of checkpoint genes also varied significantly among the clusters, with several checkpoint genes differentially expressed among the C1, C2, and C3 clusters. These findings may reflect differences in the ability of each subtype to regulate the immune response, potentially contributing to distinct treatment responses. This observation merits additional research, as manipulation of these checkpoint pathways is a promising therapeutic approach for many cancers, including in breast cancer [99]. Prediction of drug sensitivity using machine learning revealed significant differences among the three clusters for almost all current anti-tumor drugs. This discovery underscored the potential of applying computational approaches to individualize cancer treatment plans [100].

The immunological microenvironment has been widely acknowledged as a critical factor in cancer progression and response to treatment [101]. Notably, lower infiltration levels of immune cells in the high-risk group were detected, suggesting an immune-excluded or immune-desert phenotype, which is typically associated with poor clinical outcomes. This was consistent with previous findings where lower tumor-infiltrating lymphocytes (TILs) predicted worse survival in BC [102,103]. Furthermore, we observed a consistent decrease in various immune cell types’ infiltration, further stressing the immune suppression scenario in the high-risk group.

This study’s observed differences in the TMB between the high- and low-risk groups could imply varied mutational landscapes, leading to differential immunogenicity. Interestingly, a higher TMB was observed in the high-risk group, indicating potentially increased neoantigen loads. Although this should theoretically promote more robust anti-tumor immune responses, the concomitant immunosuppressive TME, evidenced by the lower immune score, could counteract this effect. This paradox echoes the “hot” versus “cold” tumor paradigm, where despite a high TMB (“hot” tumors), an immune-excluded or desert phenotype (“cold” tumors) limits a robust immune response [104].

Additionally, distinct risk groups showed differences in specific gene mutations. For instance, TP53 mutations were more prevalent in the high-risk group. As a pivotal tumor suppressor gene, TP53 mutations have been linked to aggressive BC subtypes and poorer survival rates [105]. Meanwhile, PIK3CA, another commonly mutated gene in BC, showed higher mutation rates in the low-risk group. PIK3CA is among one of BC’s most prevalent somatic mutation genes and is often associated with endocrine therapy resistance. PIK3CA mutations typically lead to PI3K exhibiting a gain-of-function and subsequently promotes tumor cell proliferation, with the prognostic value remaining contentious [106].

The built prognostic signature theoretically outperformed the traditional prognostic indicators, such as age and stage, indicating its potential clinical utility. Consistent with our findings, several studies have reported the prognostic value of lncRNA signatures in various cancer types [107,108]. Notably, this work emphasized the potential implications of pyroptosis-related lncRNAs in BC prognosis and may provide novel insights into BC pathogenesis and therapy.

In addition, the current study revealed a pyroptosis-related ceRNA regulatory network in breast cancer, further enhancing our understanding of the underlying mechanisms of breast cancer development and progression and suggesting novel potential therapeutic targets for the disease.

A pyroptosis-related mRNA–lncRNA–miRNA network was established in this study, and four clusters and ten hub genes were identified using MCODE and CytoHubba, respectively. The predicted clusters contained 11 lncRNAs and 14 miRNAs, respectively, suggesting their essential involvement in breast cancer pathogenesis through pyroptosis regulation. Indeed, previous studies have revealed the roles of these lncRNAs in cancer and supported this prediction. For example, ADAMTS9-AS1 was reported to inhibit the invasion and proliferation of BC cells via binding miR301b-3p [109]. MIR31HG was found to promote BC cell proliferation, migration, and invasion through regulation on POLDIP2 [110]. MIR22HG inhibited the progression of BC via the stabilization of the tumor suppressor gene LATS2 [111]. HOTAIRM1 promotes tamoxifen resistance in ER+ BC cells [112]. The central role of these lncRNAs in the ceRNA network of our study underscored their potential as therapeutic targets, and the potential interactions with target miRNAs were also indicated.

Interestingly, our study also revealed hub genes (MEG3, MIAT, IRF1, ADAMTS9-AS2, TP63, NOD2, NOD1, NLRP3, MAGI2-AS3, and PVT1, respectively) within the pyroptosis-related ceRNA network, some of which have been well-studied in the field of cancer biology. For instance, MEG3 is a tumor suppressor in many cancers [41,48,111], while NLRP3 regulates inflammation and cell death, which play crucial roles in tumorigenesis [26,48,112]. MIAT acts as a tumor-promoting gene and exerts essential roles in the cell cycle, immune infiltration, and in responding to estrogen in BC [46,113,114]. Their identification as hub genes in this study might provide further theoretical evidence for their significant roles in cancer.

The potential target genes predicted by the intersection of pyroptosis-related DEGs and DElncRNAs were found to be enriched in several critical biological processes and signaling pathways associated with cancer, including the “PI3K-akt signaling pathway”, “microRNAs in cancer”, and the “MAPK signaling pathway”. These pathways have been well-established in cancer pathogenesis, thereby substantiating the relevance of our ceRNA network to breast cancer and strengthening the reliability of this analysis [115,116,117].

Our work, however, is not without limitations. Firstly, the findings derived from the bioinformatics analysis should be validated experimentally both in vitro and in vivo. Additionally, while we delved into the potential roles of these lncRNAs in BC prognosis, their exact molecular mechanisms, particularly regarding their interaction with pyroptosis and immune responses, remain to be investigated. Future studies should also explore potential strategies to modulate these lncRNAs for therapeutic purposes.

In summary, we established a pyroptosis-related lncRNA signature with promising prognostic value in BC. Moreover, by integrating TME analysis and the built signature, we identified distinct BC subtypes with different immune profiles and potential responses to immunotherapy. We also established a pyroptosis-related mRNA–LncRNA–miRNA network, and many genes in this network have already been identified to exert essential roles in cancer pathogenesis. These findings underline the intertwined roles of lncRNAs, pyroptosis, and immunity in BC progression, and may open new avenues for BC management.

## 5. Conclusions

In conclusion, this study contributed to a novel understanding of pyroptosis-related lncRNA signatures and their potential as prognostic biomarkers in breast cancer. Our innovative approach encompassed a comprehensive analysis of differentially expressed genes, immune cell proportions, and checkpoint gene expression, revealing a unique biological landscape between the high- and low-risk breast cancer patients. Unraveling the complex interactions within the pyroptosis-related ceRNA network of breast cancer provided innovative insights into the molecular mechanisms underlying this disease.

One of the foremost novelties of our work was the illumination of the biological discrepancies among the breast cancer subtypes based on the pyroptosis-related lncRNA signature. This exploration not only emphasized the complexity and heterogeneity of breast cancer, but also accentuated the potential for personalized medicine based on risk score clustering, underscoring a pivotal direction for future cancer management.

However, we acknowledged the limitations of our study. Our conclusions were based on bioinformatic analyses of existing datasets and thus require further experimental validation. The intricate interplay between the lncRNAs, miRNAs, and mRNAs in the pyroptosis-related ceRNA network, while elucidated in our study, would benefit from additional studies to elucidate the precise mechanisms in greater detail.

From a broader perspective, our findings underlined the critical role of the tumor microenvironment and its relationship with prognosis. This could pave the way for the future exploration of personalized treatments, driving a paradigm shift towards tailored therapeutic strategies. Despite the limitations mentioned, we believe our study stands as a theoretical cornerstone that could spur further research and innovation in breast cancer therapy.

Future work should focus on validating these bioinformatic findings and translating them into viable clinical applications, potentially leading to breakthroughs in therapeutic strategies for breast cancer.

## Data Availability

This data can be found at https://portal.gdc.cancer.gov.

## Supplementary Materials

The following supporting information can be downloaded at: https://www.mdpi.com/article/10.3390/life13071599/s1, Figure S1: K–M analyses of overall survival and the proportion of multiple immune cells calculated using various software tools; Figure S2: Drug sensitivity of the high- and low-risk groups to parts of drugs; Figure S3: Drug sensitivity of the C1, C2, and C3 clusters to parts of drugs.
