# Supplementary material for: Bridging Pyroptosis and Immunity: A Comprehensive Study of the Pyroptosis-Related Long Non-Coding RNA Signature in Breast Cancer
Source: Life (Basel). 2023 Jul 21;13(7):1599. doi: 10.3390/life13071599 (PMC10381440; doi:10.3390/life13071599)
Supplement: Supplementary file 1 [file life-13-01599-s001.zip › life-2464255-supplementary.pdf]

life

MDPI

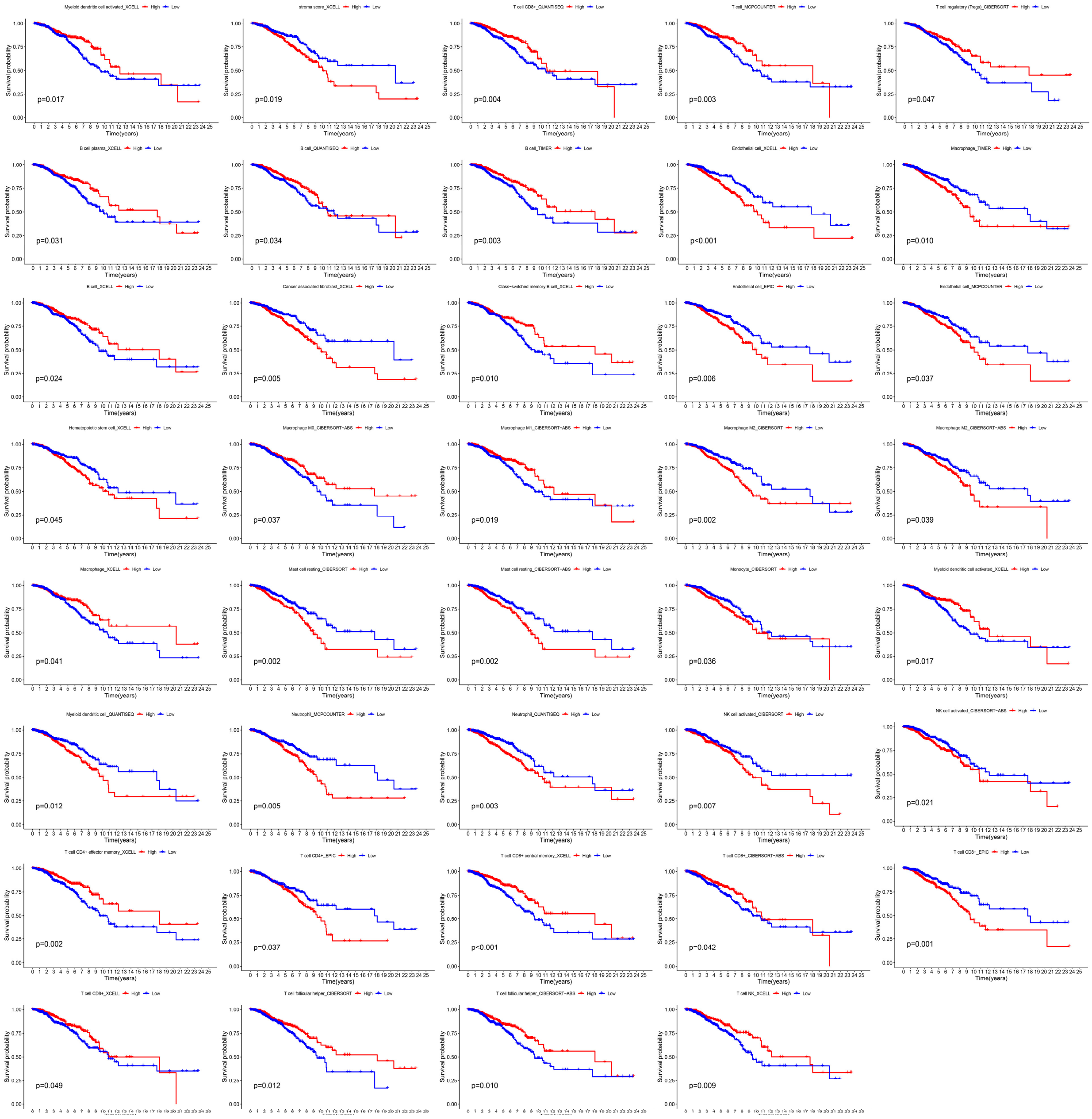

Figure S1. K–M analyses of overall survival and the proportion of multiple immune cells calculated using various software tools.

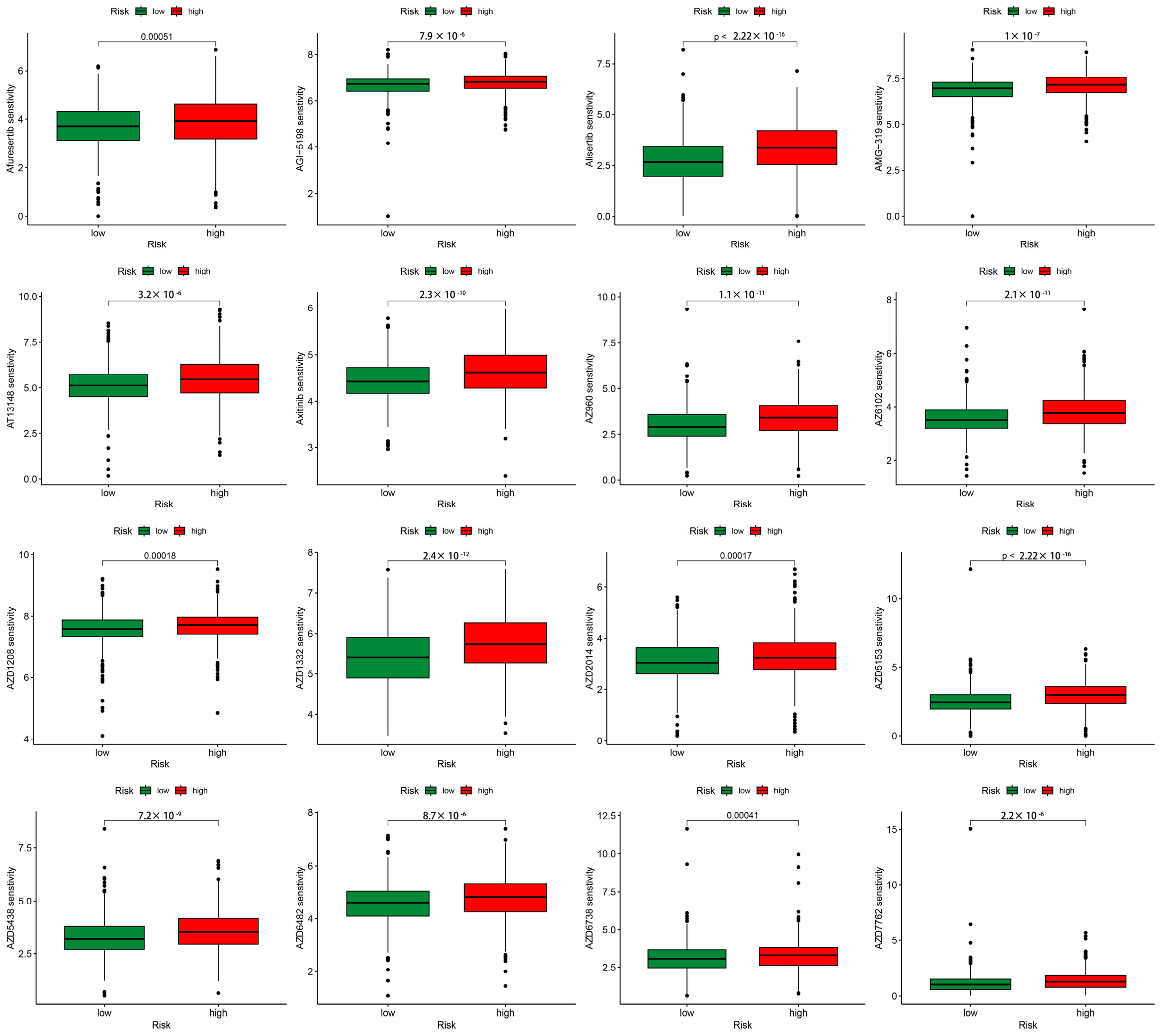

Figure S2. Drug sensitivity of the high- and low-risk groups to parts of drugs.

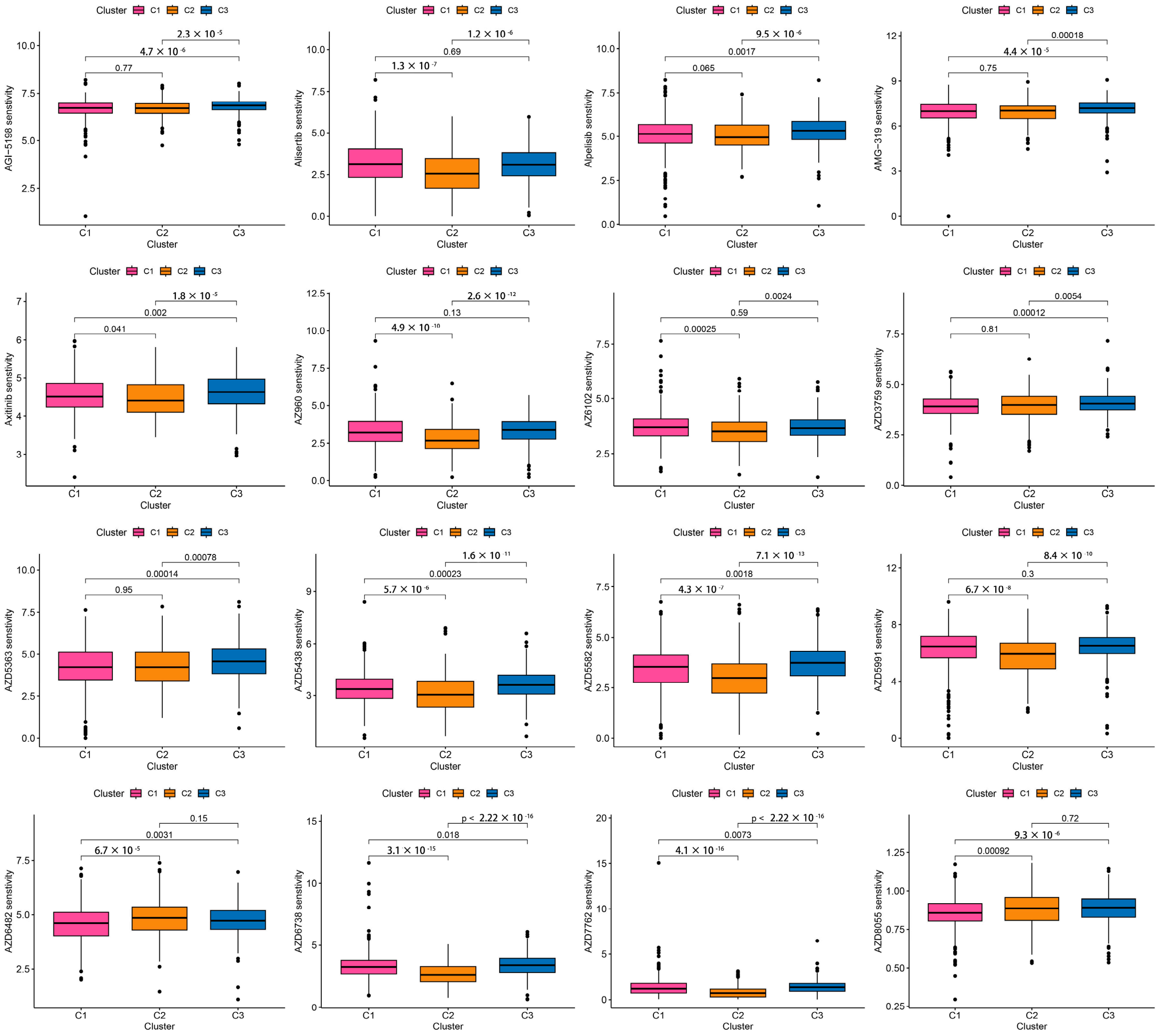

Figure S3. Drug sensitivity of the C1, C2, and C3 clusters to parts of drugs.
